# The Sanitary Condition of Buffalo

**Published:** 1879-09

**Authors:** 


### EDITORIAL.

---

### THE SANITARY CONDITION OF BUFFALO.

THE importance of observing, and if possible controlling the causes which produce sickness and excessive mortality, has always been recognized by the profession, and the JOURNAL will speak with no uncertain sound upon such topics as are connected with the health and well-being of the community. One of the hopeful signs of the times is the widely extended interest in public health questions, and the organization of competent sources of advice and authority in matters affecting the protection of life and health. The movement is but in its infancy, but the influence of all well directed efforts for promoting sanitary knowledge and its application, takes hold upon the highest interests of mankind, and will, in the advancing civilization and higher culture of communities, become more and more a matter of organized and wise administration.

The publication, by the National Board of Health, of mortality statistics from the chief cities of the world, is an important step in this direction. Their report shows not only the annual death rate per 1000 in each city, but also the number of deaths resulting from the principal diseases thereby, giving much valuable information to the sanitarian and vital statistician.

We note, with regret, the series of blanks usually found after the name of Buffalo in these reports. The value of such statistical knowledge is largely dependent upon their completeness.

In our present issue we give a mortality table, condensed from the reports in the National Board of Health Bulletin, and we propose to continue them in the future. We trust that reports from Buffalo will regularly appear, and we have reason to believe that the comparison would be highly favorable to our own city. Certainly the natural advantages which Buffalo possesses would place it at the head of the list if proper sanitary regulations are enforced.

It is to the physicians that we must chiefly look, to make the knowledge of sanitary science, possessed by the educated, serviceable to the protection of the people—and in this duty the members of the Buffalo Medical Association have not been behind their professional brethren of other cities. At the last meeting of the American Medical Association, Prof. T. F. Rochester read a paper on the "Limitation and Prevention of Epidemic Diseases," which attracted much favorable attention.

The paper has been reported in full in many of our best medical journals, and is looked upon as a valuable contribution to the medical and sanitary literature of the day.

At a late meeting of the Buffalo Medical Association, a paper was read by Dr. A. R. Davidson on the "Sanitary Chemistry of Water," which is of peculiar interest to us, as a large part of it was devoted to the water supply of our own city. As the paper was published in full in the daily press, it will not be necessary to give it a place in our columns, but we desire to call attention to a few of the points therein presented:

That many of our city wells are contaminated, and that much sickness is dependent upon the use of impure water has been a fact recognized by the practical experience of all of our physicians. The marked diminution of disease, since the introduction of pure Niagara water—on the completion of the tunnel—has also been generally admitted. But a considerable portion of our city is not, as yet, supplied with Niagara water, and it is in that part of the city that Dr. Davidson most often finds the water of the wells bad, and sometimes so *horribly bad* as to indicate direct connection with leaky sewer and privy vault.

That such a condition of things should be allowed to remain uncorrected is shameful. If the wealthier portion of the city had to depend upon such a water supply, no time would be lost in taking measures to obtain a pure water at any cost; but, as the districts dependent upon these wells are populated chiefly by the poor and laboring classes, who cannot afford to pay for Niagara water, the question of what to do? seems to have given our City Fathers much trouble to decide. At a late meeting of the Common Council, however, action was taken, which gives some promise of relief, and we hope soon to be able to chronicle the fact that an abundant supply of pure water is within the reach of every citizen of Buffalo.

At the July meeting of the Buffalo Medical Association, Prof. James P. White called attention to the filthy condition of many of the public streets—a condition which is by no means improved by the practice of sprinkling.

The passage of the sprinkling cart, while it "lays the dust," makes mud which is equally annoying to the pedestrian.

Dr. White's objections were, however, taken from a sanitary point of view. He considered that the rapid decomposition of the heterogeneous materials, which go to make up street mud, produce an unhealthy condition of the atmosphere, and was injurious to the public health. However that may be, the plan proposed to correct the trouble is one that will commend itself to every sensible mind. It was that the streets should be clean, and kept clean, by the constant application of the street cleaner's broom, and he insists that the expense of keeping the streets perfectly clean in this way will not exceed the cost of sprinkling. In view of the importance of the subject, to which the Doctor had called attention, a special committee was appointed by the Association to investigate and report at a subsequent meeting.

